# Immersive Virtual Reality eHealth Intervention to Reduce Anxiety and Depression in Pregnant Women: Randomized Controlled Trial

**DOI:** 10.2196/71708

**Published:** 2025-04-30

**Authors:** Marta Jimenez-Barragan, Amparo Del Pino Gutierrez, Gloria Sauch Valmaña, Olga Monistrol, Carme Monge Marcet, Mar Pallarols Badia, Ignasi Garrido, Anna Carmona Ruiz, Oriol Porta Roda, Cristina Esquinas, Gemma Falguera Puig

**Affiliations:** 1 Department of Public Health, Mental Health and Maternal and Child Health Nursing Faculty of Nursing Universitat de Barcelona Barcelona Spain; 2 Health Promotion in Rural Areas Research Group, Gerència Territorial de la Catalunya Central, Institut Català de la Salut Institut Universitari per la Recerca a l’Atenció Primària de Salut Jordi Gol i Gurina (IDIAPJGol) Institut Català de la Salut Barcelona Spain; 3 Departament of Nursing and Physiotherapy, Campus Igualada Universitat de Lleida Lleida Spain; 4 Department of Gynecology and Obstetrics, Assistential Foundation Mútua Terrassa Mútua Terrassa Terrassa Spain; 5 Psychiatry and Mental Health Service. Eating Disorder Unit Mútua Terrassa Terrassa Spain; 6 Department of Obstetrics and Gynaecology Fundacio Assitencial Mollet Mollet Spain; 7 Department of Gynecology and Obstetrics Mútua Terrassa Terrassa Spain; 8 Primary Care Group, Germans Trias i Pujol Research Institute (IGTP) Research Group Atenció a la Salut Sexual i Reproductiva (GRASSIR), Institut Universitari d'Investigació en Atenció Primària Jordi Gol (IDIAP Jordi Gol) Institut Català de la Salut Barcelona Spain

**Keywords:** virtual reality, eHealth, pregnancy, mental health, anxiety, depression, randomized controlled trial, antenatal care

## Abstract

**Background:**

Mental health during pregnancy is a critical factor influencing maternal and fetal outcomes. Anxiety and depression affect up to 30% of pregnant women, with significant consequences for maternal well-being and child development. Despite this, interventions during pregnancy remain limited, creating a need for innovative, accessible solutions.

**Objective:**

This study aimed to evaluate the effectiveness of an immersive virtual reality (IVR) eHealth intervention in reducing anxiety and depression symptoms in women during pregnancy.

**Methods:**

A 2-arm, randomized controlled trial was conducted across 5 primary care centers in Catalonia, Spain, between October 2021 and May 2024. The study included pregnant women (N=70) aged ≥18 years with moderate anxiety and depression symptoms (Edinburgh Postnatal Depression Scale [EPDS] scores: 9-12) at 12 to 14 weeks of gestation. They were randomly assigned (1:1) to an IVR intervention or standard care group. The intervention group engaged in daily 14-minute IVR mindfulness and relaxation sessions for 6 weeks. Mental health outcomes were assessed using the EPDS and State-Trait Anxiety Inventory.

**Results:**

The intervention group demonstrated significant reductions in EPDS scores, with a mean decrease from 11.32 (SD 0.96) to 7.25 (SD 1.32; *P*<.001), compared to an increase in the control group from 11.32 (SD 0.94) to 16.23 (SD 1.25; *P*<.001). Similarly, State-Trait Anxiety Inventory scores improved markedly in the intervention group (mean decrease from 57.94, SD 5.23 to 35.03, SD 6.12; coefficient –30.47, 95% CI −45.23 to −15.72; *P*<.001), while the control group experienced a nonsignificant increase (from 66.10, SD 5.89 to 72.91, SD 6.34; *P*=.68). High adherence rates were observed, with 79% (26/33) of participants completing ≥30 sessions. Participant satisfaction was high, with 87% (29/33) reporting being “very satisfied” with the intervention.

**Conclusions:**

The IVR eHealth intervention significantly reduced symptoms of anxiety and depression, demonstrating its potential as an accessible and effective tool for mental health support during pregnancy. High adherence and satisfaction levels underscore its feasibility and acceptability. Future research should explore the long-term effects and scalability of IVR interventions in diverse settings.

**Trial Registration:**

ClinicalTrials.gov NCT05756205; https://clinicaltrials.gov/study/NCT05756205

**International Registered Report Identifier (IRRID):**

RR2-10.1186/s12912-023-01440-4

## Introduction

### Background

Mental health during pregnancy is a crucial aspect of women’s overall well-being, influencing not only the mother but also the development of the fetus and the subsequent outcomes for the child [1,2]. Up to 30% of women may experience mental health disorders during pregnancy, including anxiety and depression [3-5]. These disorders, often interrelated, have a significant impact not only on the mother’s health but also on the neurological development of the fetus, potentially leading to complications, such as preterm birth, low birth weight, and long-term emotional difficulties [6]. These adverse effects not only affect the immediate postpartum period but can also influence the child’s long-term development, with possible repercussions on behavior, cognition, and mental health [7].

Despite their importance, interventions to address mental health disorders during pregnancy have been limited, with many focusing on the postpartum period, leaving a critical need for attention during pregnancy itself [8]. This deficit in intervention during pregnancy is concerning, as early detection and appropriate treatment of these disorders are essential to ensuring optimal health outcomes for both the mother and the baby [9]. Inadequate treatment or lack of interventions may increase the risk of obstetric complications, such as preterm birth or low birth weight, as well as long-term emotional and behavioral problems in the child [10,11].

Low-intensity interventions, as described by the National Institute for Health and Care Excellence, are particularly important for women experiencing mild to moderate symptoms of anxiety and depression. The National Institute for Health and Care Excellence recommends these interventions as a first step before considering more intensive treatments, as they can provide effective support with fewer adverse effects and are accessible to a wide population [12]. In this context, digital health technologies, known as eHealth, offer a promising new avenue for improving access to care and supporting the mental health of pregnant women [13].

The use of eHealth technologies is growing exponentially worldwide, supported by guidelines from international organizations, such as the World Health Organization, which promote the use of these technologies to improve public health [14]. According to World Health Organization, eHealth technologies can play a key role in expanding access to mental health services [15]. Their application in perinatal mental health has shown promising results in preliminary studies, especially in reducing symptoms of anxiety and depression [16]. These technologies offer the possibility of providing low-intensity interventions, such as mindfulness, relaxation, and other stress management techniques, in a way that is accessible and convenient for women [17]. In addition, mobile apps allow for closer and more personalized monitoring of women’s mental health, offering continuous support and enabling quicker interventions in the event of concerning symptoms [18]. eHealth interventions can be a flexible alternative for those women who, for various reasons, cannot easily access traditional mental health services, offering solutions tailored to their individual needs and daily routines [19].

In this context, immersive virtual reality (IVR) is emerging as an innovative tool in the health field. IVR allows users to immerse themselves in simulated environments in a fully immersive manner, offering therapeutic experiences that can be highly effective in managing anxiety, depression, and other mental disorders [20]. Several studies have demonstrated that IVR can help reduce stress and improve emotional well-being in patients, providing cognitive and emotional distraction that facilitates relaxation and symptom reduction [21]. Moreover, its ability to recreate controlled environments makes it useful for exposure therapy and other psychological interventions [22]. This technology, combined with other eHealth tools, can offer personalized and highly effective solutions for pregnant women experiencing mental health issues, opening new possibilities for their management and treatment [23].

Furthermore, the integration of eHealth and mobile health technologies is gaining traction as an effective means of delivering mental health interventions. These technologies provide a direct, low-cost, and engaging manner to deliver care, making mental health support more accessible [24,25]. The global increase in the use of these technologies has led to a growing body of research supporting their efficacy, particularly in treating mood disorders during pregnancy [26,27]. Recent studies have also underscored the importance of mobile health interventions specifically designed for pregnant women, highlighting their effectiveness in reducing anxiety and depression symptoms during pregnancy [5,28].

### Objectives

The objective of this study was to evaluate the effectiveness of an IVR eHealth intervention in improving mental health outcomes during pregnancy, specifically by comparing symptoms of anxiety and depression between pregnant women in the intervention and control groups, assessing satisfaction with antenatal care between the 2 groups, and monitoring adherence to the intervention by tracking the number of completed IVR sessions.

## Methods

### Study Design

This study was a 2-arm, prospective, randomized controlled trial (RCT) designed to assess the efficacy of a low-intensity eHealth intervention in improving mental well-being among pregnant women. The CONSORT (Consolidated Standards of Reporting Trials) guidelines for RCTs were followed. Participants were randomly assigned (1:1) to the intervention or control group using a computer-generated randomization sequence. Specifically, the Epidat (version 4.2; Epidemiology Service of the Dirección Xeral de Saúde Pública, Xunta de Galicia) software was used to generate the randomization list, ensuring allocation concealment and minimizing selection bias. To maintain the integrity of the randomization process, the allocation sequence was generated independently by the principal investigator (MJ-B) before recruitment. Randomization was stratified by recruitment center to control potential site-specific variations. Allocation was concealed from participants and researchers responsible for data collection to reduce potential biases. Participants and health care providers were masked to group allocation to the extent possible in an intervention of this nature.

The study included pregnant women aged ≥18 years who attended primary care centers affiliated with the sexual and reproductive health care (*Atenció a la Salut Sexual i Reproductiva* [ASSIR; Sexual and Reproductive Health Care in English]). It was conducted across 5 primary care centers (ASSIR) in Catalonia, Spain, between October 2021 and May 2024. Between 12 and 14 weeks of gestation, participants who scored between 9 and 12 on the Edinburgh Postnatal Depression Scale (EPDS)—validated Spanish version [29]—were recruited and randomly allocated to either the intervention group, which received the eHealth intervention, or the control group, which received standard care. Participants were also screened for mental health conditions using the State-Trait Anxiety Inventory (STAI)—validated Spanish version [30]. Those with a diagnosis of severe psychiatric disorder, ongoing treatment by mental health specialists, and a history of gender-based violence were excluded.

### Procedure

Participants who scored between 9 and 12 on the EPDS, indicating symptoms of moderate anxiety and depression, were considered for inclusion in the study. Participants were randomly assigned (1:1) to the intervention or control condition. The principal investigator generated a randomization list using the Epidat (version 4.2) software, and all study participants were masked ensuring the randomization process. Women assigned to the control group were informed that their follow-up would proceed as usual, while those in the intervention group received additional information about the eHealth intervention, including the use of IVR.

### Intervention

**Figure 1:**
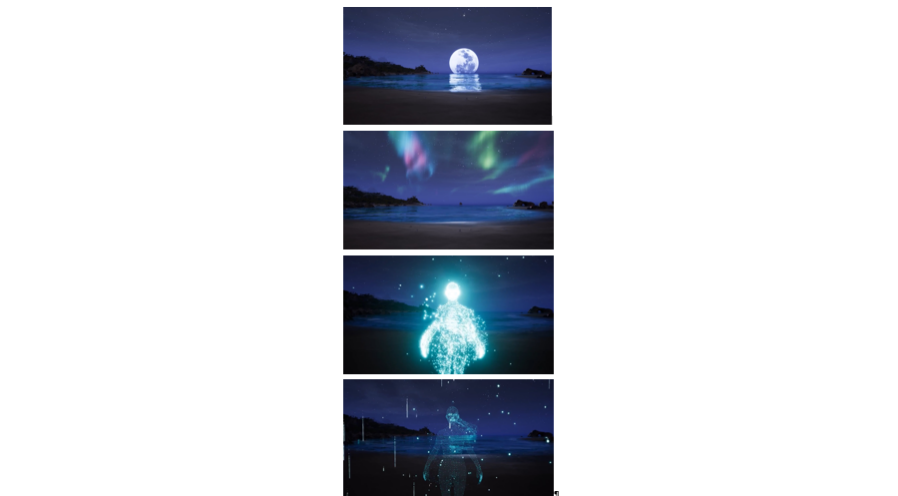
Screenshots of the virtual reality experience.

**Figure 2:**
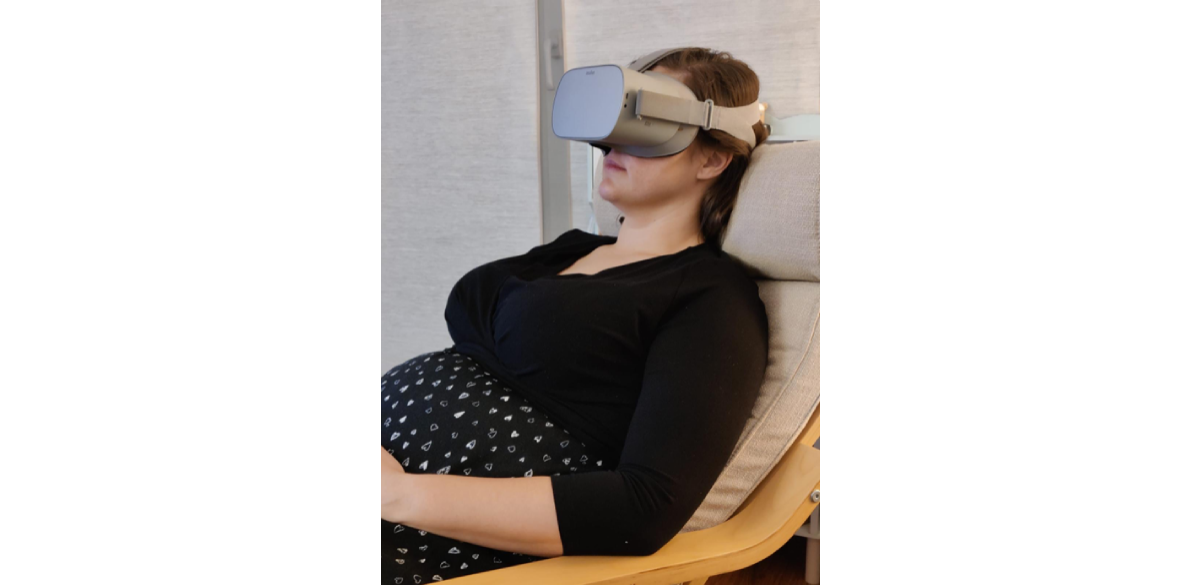
A pregnant woman using the immersive virtual reality application at home (with written patient permission).

The intervention group received an eHealth-based mental health program, which included the use of IVR technology to deliver mindfulness and relaxation exercises designed to reduce anxiety and depression during pregnancy. The IVR intervention lasted 14 minutes per session and was performed daily for 6 weeks. These sessions were based on mindfulness techniques, conscious breathing, and progressive muscle relaxation, with the aim of providing an immersive therapeutic environment that would promote stress reduction and improve the emotional well-being of the participants. The content of the intervention was developed by Delaguila Games [31], a company specializing in virtual reality applied to health care (Figure 1). The sessions were designed to be self-guided and accessible from home (Figure 2), using an Oculus Go (Meta Reality Labs) virtual reality headset. The program contained 3 main modules that could be selected individually or together, adapting to the needs and preferences of each participant. To ensure adherence and effectiveness of the intervention, the total number of sessions completed by each participant was recorded.

### Control

Participants in the control group received the usual antenatal care provided by the public health system. This refers to the pregnancy follow-up protocol established by the Department of Health of the Generalitat de Catalunya, which outlines standard guidelines for prenatal care and monitoring [32].

### Sample

Pregnant women aged ≥18 years who were followed at the primary care centers of sexual and reproductive health care (ASSIR) of Mútua Terrassa in Barcelona, Spain, were invited to participate in the study.

In a previous published interventional work [33] using a mindfulness program to evaluate changes in the EPDS score in pregnant women, the authors reported a mean difference of −2.56 (SE 0.72) between the 2 groups of study (intervention and control) after the program. From that, the SD was calculated as SE=SD/Root(n), so SD=SE × Root(n). In this case, as the n was different in both groups (51 and 45), we used the minimum of both (n=45), obtaining a joint SD of 4.83. Assuming a power of 80% to detect differences in contrast of the null hypothesis H_0_: μ_1_=μ_2_ using a bilateral Student *t* test for 2 independent samples, a significance level of 5%, and assuming a mean difference between both groups of −3.5 (SD 4.83 units), it was necessary to include 31 women in the nonintervention group and 31 women in the intervention group, totaling 66 patients in the study. Considering that the expected drop-out rate is 5%, it was necessary to recruit 70 women (35 in each group).

### Measures

#### Primary Outcome: Mental Health Status

The mental health status of the participants was measured using the EPDS and the STAI. The EPDS is a self-report scale comprising 10 items, where participants choose 1 of 4 responses that best describes how they felt during the past week. The EPDS has been validated in Spanish and shows good sensitivity (79%) and specificity (95.5%), with a Cronbach α of 0.87 [29]. Scores range from 0 to 30, with scores ≥13 indicating a positive screen for depression. The STAI assesses 2 dimensions of anxiety: state anxiety (a temporary condition in response to a specific situation) and trait anxiety (a general tendency to perceive situations as threatening) [30]. Both scales have demonstrated robust psychometric properties in various populations.

#### Secondary Outcomes

##### Satisfaction With Pregnancy Care

Satisfaction with pregnancy care was measured using a validated self-completed questionnaire [34], consisting of 28 items that assess different aspects of care, including equipment, accessibility, organization of the consultation, and the competence of the staff. The scale uses a 5-point Likert system, and scores are categorized as satisfied (scores >3) or very satisfied (scores >4). The reliability of this tool has been demonstrated with a Cronbach α of 0.92 [35].

##### Adherence to the Intervention Protocol

Adherence to the intervention protocol was monitored by tracking the number of sessions completed by each participant in the intervention group. In addition, participants in this group were asked to complete a questionnaire evaluating the usability and accessibility of the eHealth technology used during the study [36]. The number of sessions and time spent on each session were recorded to assess adherence and participant engagement with the intervention.

##### Pulse Measurements

Pulses were recorded before and after the intervention.

Participants were encouraged to report any adverse events, discomfort, or unexpected side effects experienced during or after the IVR sessions. If the participant experienced dizziness, nausea, or an increase in anxiety symptoms, she was instructed to stop the session and rest. If symptoms persisted, participants could contact the research team via an email address specially designed to resolve any problems. Participants had the right to withdraw from the study at any time, without having to justify their decision, and without this affecting their regular prenatal follow-up. In addition, the study team, made up of midwives and mental health professionals, was available to provide additional psychological support if necessary, ensuring that participants felt safe and supported throughout the intervention.

### Data Collection and Management

Data were collected at 2 time points: baseline (12-14 weeks of gestation) and 6 weeks after the start of the intervention. All data were securely stored and anonymized, with access restricted to authorized personnel only. Participants’ personal information was protected according to the ethical standards of Mútua Terrassa.

### Statistical Analysis

Qualitative variables were described using absolute frequencies and percentages. Quantitative variables were summarized using the mean, SD, and median. The Kolmogorov-Smirnov test was used to assess the normality of distributions.

Sociodemographic and clinical characteristics were compared across groups. For quantitative variables, Mann-Whitney *U* tests were conducted. The chi-square test (or Fisher exact test for frequencies <5) was used to compare categorical variables. The effectiveness of the intervention was evaluated using mixed-effects models to assess changes in mental health outcomes over time by group. The interaction between time and intervention, as well as potential confounding factors such as age, previous admissions, current planned pregnancy, and personal history of anxiety, were also considered in these analyses.

In addition, the postintervention scores of the EPDS and STAI-E were transformed into binary variables. For EPDS, a cutoff score of 9 was selected based on bibliographic criteria [29], while for STAI-E, the third quartile value (>86 points) was used [30]. Finally, several logistic regression models were conducted for the primary outcomes with the study group included as an independent variable. These models were also adjusted for the previously mentioned confounding factors, including preintervention EPDS and STAI-E scores. Hosmer-Lemeshow goodness-of-fit tests were performed to assess the overall fit of the models [37]. Receiver operating characteristic curves were also calculated for this approach.

Statistical significance was set at *P*<.05, and all analyses were performed using R version 4.2.1 (R Foundation for Statistical Computing). For the mixed-effects models, we used the *nlme* package with the *lme* function and the *stats* package with the *glm* function in the logistic models.

### Ethical Considerations

The study protocol was approved by the Drug Research Ethics Committee of Mútua Terrassa (B1803). Informed consent was obtained from all participants before their inclusion in the study. The study was conducted in accordance with the Declaration of Helsinki and relevant European Union regulations on data protection. The informed consent process was conducted following strict ethical guidelines to ensure that all participants fully understood the nature of the study and its potential risks and benefits, and that their participation was voluntary. The steps taken for the informed consent process are provided in Textbox 1.

These measures were taken to guarantee that all participants provided truly informed and voluntary consent, minimizing any risk of coercion or undue influence.

**Informed consent process.**

- A comprehensive explanation: before the enrollment, all participants received a detailed explanation of the study’s objectives, procedures, potential benefits, and possible risks. This was done through both written and verbal communication by trained health care professionals.
- Time for reflection and questions: participants were given sufficient time to read the informed consent document and ask any questions. Researchers provided clarifications to ensure that participants had a clear understanding before signing the consent form.
- Assessment of understanding: to ensure comprehension, participants were asked to summarize key aspects of the study in their own words. If any misunderstandings were identified, additional explanations were provided.
- Voluntary participation and withdrawal rights: it was explicitly stated that participation was entirely voluntary, and that participants could withdraw at any time without any consequences for their health care or treatment.
- Safeguards against coercion: to prevent undue influence, recruitment was conducted by independent health care professionals who were not involved in participants’ routine medical care. No financial or material incentives were provided to encourage participation.
- Ethics approval: the study protocol, including the informed consent procedures, was reviewed and approved by the Drug Research Ethics Committee of Mútua Terrassa (B1803), ensuring compliance with ethical and legal standards.

## Results

### Overview

**Figure 3:**
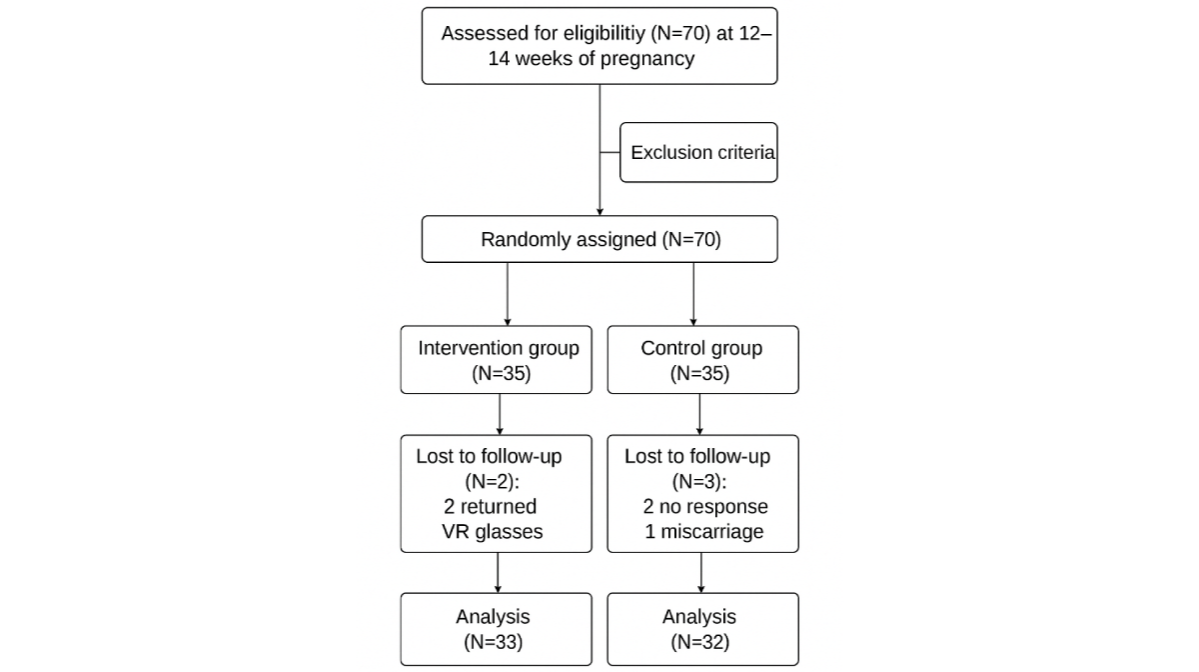
CONSORT (Consolidated Standards of Reporting Trials) participant flowchart for the randomized controlled trial. VR: virtual reality.

The study included 70 participants with a mean age of 31.9 (SD 4.83) years, evenly distributed between the intervention group (n=35, 50%) and the control group (n=35, 50%; Figure 3). There were 2 losses to follow-up in the intervention group and 3 in the control group. All cases of loss to follow-up occurred in the period after the intervention.

### Sociodemographic and Clinical Characteristics

Regarding sociodemographic and clinical characteristics, the intervention group had a higher mean age (in years) compared with the control group (33.77*,* SD 3.46 vs 30.03, SD 5.3; *P*=.003). Furthermore, a significantly higher proportion of participants in the intervention group reported having their own income (35/35, 100% vs 21/35, 60%; *P*<.001) and a university-level education (24/35, 69% vs 8/35, 23%; *P*<.001*;* Table 1).

**Table 1:** Comparison of sociodemographic and clinical characteristics between the control and intervention groups (N=70).

Characteristic			Total (N=70)		Control group (n=35)		Intervention group (n=35)		*P* value
Age (y), mean (SD)			31.9 (4.83)		30.03 (5.3)		33.77 (3.46)		.003^a^
**Country of origin, n (%)**									
	Spain	50 (71)		17 (49)		33 (94)		<.001^b^	
	Morocco	10 (14)		10 (29)		0 (0)		—^c^	
	Other countries	10 (14)		8 (23)		2 (6)		—	
**Religious affiliation, n (%)**									
	Christian	21 (30)		9 (26)		12 (34)		.001^b^	
	Muslim	10 (14)		10 (29)		0 (0)		—	
	Atheist	36 (51)		15 (43)		21 (60)		—	
	Other	3 (4)		1 (3)		2 (6)		—	
**Educational level**, **n (%)**									
	University	32 (46)		8 (23)		24 (69)		<.001^b^	
	Secondary	30 (43)		19 (54)		11 (31)		—	
	Primary	6 (9)		6 (17)		0 (0)		—	
**Employment status** **, n (%)**									
	Specialized worker	37 (53)		11 (31)		26 (74)		<.001^b^	
	Seeking employment	12 (17)		7 (20)		5 (14)		—	
	Other occupations	21 (30)		17 (49)		4 (11)		—	
**Personal income**, **n (%)**									
	Yes	56 (80)		21 (60)		35 (100)		<.001^b^	
	No	14 (20)		14 (40)		0 (0)		—	

^a^*P* value results comparing the study groups by Mann-Whitney *U* test are considered statistically significant when <.05.

^b^*P* value results comparing the study groups by chi-square tests are considered statistically significant when <.05.

^c^Not available.

Regarding prepregnancy mental health treatment history, participants who reported treatment history had received a variety of interventions, including individual or group psychotherapy, pharmacological treatment with antidepressants or anxiolytics, and follow-up by mental health professionals in primary or specialist health care. The length of treatment varied among participants, ranging from brief interventions <6 months to continued treatment >2 years. Disaggregated data about specific treatment adherence or the exact type of medication used by each participant were not available in this study.

Regarding obstetric characteristics, 86% (30/35) of pregnancies in the intervention group were planned, compared with 57% (20/35) in the control group (*P*=.02; Table 2). No significant differences were found between the groups regarding the history of cesarean sections, traumatic deliveries, or high-risk pregnancies (all *P*>.05).

**Table 2:** Clinical and obstetric characteristics.

Category and characteristic		Total (N=70), n (%)	Control group (n=35), n (%)	Intervention group (n=35), n (%)	*P* value^a^
**Clinical**					
	Current smoker	10 (14)	7 (20)	3 (9)	.31
	Mental health treatment before pregnancy	40 (57)	15 (43)	25 (71)	.03
	Family history of psychiatric disorders	22 (31)	12 (34)	10 (29)	.80
	Planned current pregnancy	50 (71)	20 (57)	30 (86)	.02
	Current pregnancy achieved through assisted reproduction	7 (10)	1 (3)	6 (17)	.11
	History of anxiety	47 (67)	18 (51)	29 (83)	.01
	History of depression	18 (26)	8 (23)	10 (29)	.78
**Obstetric^b^**					
	Previous miscarriage	18 (26)	7 (20)	11 (31)	.41
	Previous cesarean section	4 (6)	2 (6)	2 (6)	.99
	Previous traumatic childbirth experience	11 (16)	5 (14)	6 (17)	.99
	High-risk current pregnancy	33 (47)	15 (43)	18 (51)	.63
	Partner involvement in care	68 (97)	33 (94)	35 (100)	.50
	Previous fetal loss	0 (0)	0 (0)	0 (0)	.99
	Method of conception: IVF^c^	7 (10)	1 (3)	6 (17)	.11
	Prepregnancy mental health diagnosis	20 (29)	8 (23)	12 (34)	.03

^a^*P* value results comparing the study groups by chi-square test are considered statistically significant when <.05.

**^b^**Gestational week of all participants at enrollment was 12 to 14 weeks.

^c^IVF: in vitro fertilization.

### Comparison of EPDS Scores Between Groups Before and After the Intervention

**Figure 4:**
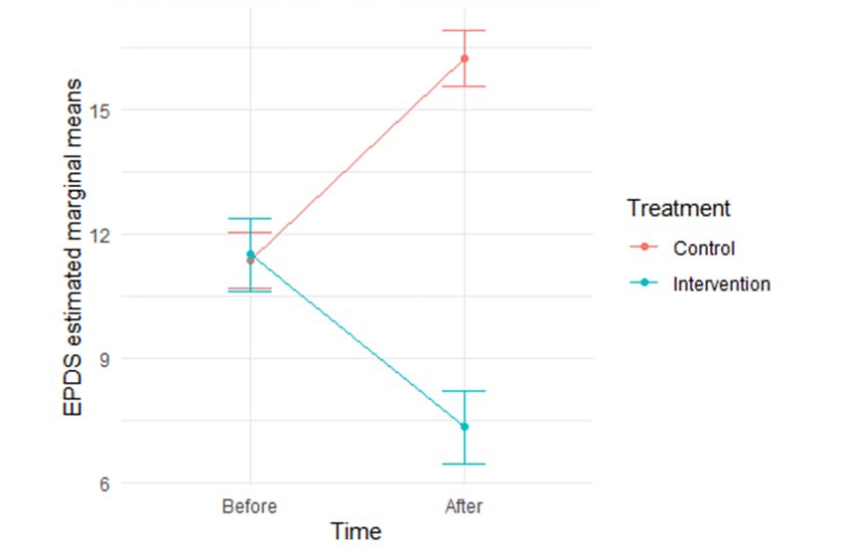
Interaction between time and treatment in the Edinburgh Postnatal Depression Scale (EPDS) score.

At baseline, the EPDS score was comparable between both groups (*P*=.56). Following the intervention, the mean EPDS score significantly decreased in the intervention group (from 11.32 to 7.25; *P*<.001), whereas it increased in the control group (from 11.32 to 16.23; *P*<.001). The adjusted mixed-effects model revealed a significant interaction between time and treatment (coefficient=−9.03, 95% CI −11.29 to −6.76; *P*<.001), indicating a substantial reduction in EPDS scores attributable to the intervention over time (Figure 4).

Furthermore, the adjusted logistic regression model demonstrated that participants in the intervention group had a significantly lower likelihood of exhibiting postintervention EPDS scores ≥9 points compared to the control group (odds ratio [OR] 0.03, 95% CI 0.01-0.8; *P*=.001; Table 3). In addition, a planned pregnancy was associated with a lower probability of achieving EPDS scores ≥9 points. In contrast, a higher preintervention EPDS score was associated with an increased likelihood of achieving EPDS scores ≥9 points (OR 3.32, 95% CI 1.52-9.58; *P*=.008). The model demonstrated good calibration, as evidenced by the Hosmer-Lemeshow goodness-of-fit test (*P*=.10) and the area under the receiver operating characteristic curve (area under curve=0.82).

**Table 3:** Multivariate logistic regression model for postintervention Edinburgh Postnatal Depression Scale (EPDS) score (>9 points).

Variable	Multivariate^a^ odds ratio (95% CI)	*P* value^b^
Intervention group	0.03 (0-0.18)	.001^b^
Preintervention EPDS score	3.32 (1.52-9.58)	.008
Age (y)	0.99 (0.8-1.12)	.93
Personal income	2.2 (0.12-41.26)	.58
Current planned pregnancy	0.1 (0.01-0.65)	.03
Personal history of anxiety	4.48 (0.66-50.4)	.16

^a^Hosmer–Lemeshow goodness-of-fit test, *P*=.10. Area under the receiver operating characteristic curve=0.82 (95% CI 0.78-0.85).

^b^*P* value is considered statistically significant when <.05.

### Comparison of STAI-E Scores Before and After the Intervention

**Figure 5:**
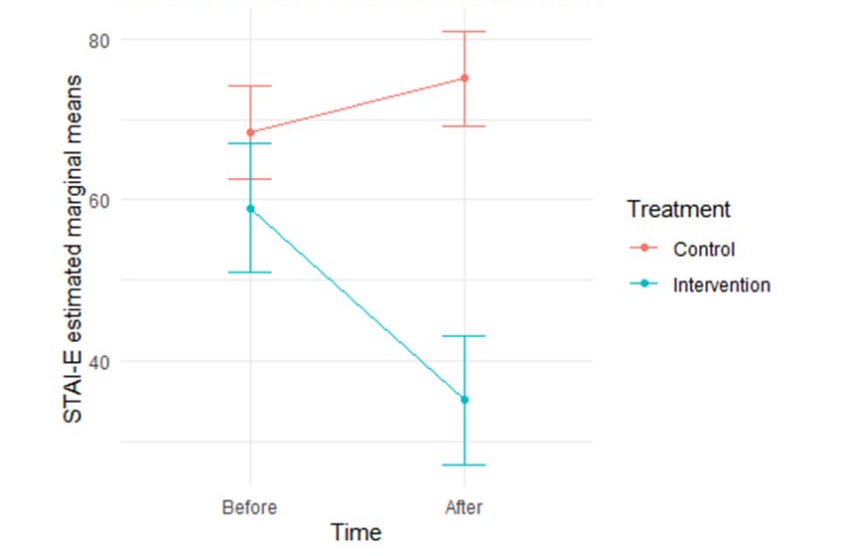
Interaction between time and treatment in the State-Trait Anxiety Inventory (STAI)-E score.

At baseline, the STAI-E score was similar in both groups (*P*=.12). Following the intervention, the intervention group experienced a significant reduction in mean scores (from 57.94 to 35.03; *P*<.001), whereas the control group showed an increase (from 66.10 to 72.91; *P*<.001). The adjusted mixed-effects model identified a significant interaction between time and treatment (coefficient=−30.47, 95% CI −45.23 to −15.72; *P*<.001; Figure 5), indicating a substantial improvement attributable to the intervention over time, similar to the effects observed with the EPDS. Conversely, the control group exhibited a nonsignificant change, with mean scores increasing slightly from 66.10 to 72.91 (*P*=.68).

Furthermore, the adjusted logistic regression model demonstrated that participants in the intervention group had a significantly lower likelihood of presenting postintervention STAI-E scores >86 points compared to the control group (OR 0.05, 95% CI 0.01-0.40; *P*=.01). In addition, a higher preintervention STAI-E score was associated with an increased probability of exceeding the threshold (OR 1.04, 95% CI 1.01-1.08; *P*=.02*;* Table 4).

**Table 4:** Multivariate logistic regression model for postintervention State-Trait Anxiety Inventory (STAI) scores (state; >86 points)

Variable	Multivariate^a^ odds ratio (95% CI)	*P* value^b^
Intervention group	0.09 (0.00-0.73)	.05^b^
Preintervention STAI-E score	1.04 (1.01-1.08)	.02
Age (y)	0.83 (0.64-1.01)	.08
Personal income	0.40 (0.05-2.46)	.34
Current planned pregnancy	0.32 (0.05-1.75)	.20
Personal history of anxiety	0.95 (0.16-6.05)	.96

^a^Hosmer–Lemeshow goodness-of-fit test, *P*=.18. Area under the receiver operating characteristic curve=0.79 (95% CI 0.65-0.88).

^b^*P* value is considered statistically significant when <.05.

### Pulse Measurements

Before intervention, the intervention group had a mean pulse of 85.91 (SD 5.47) beats per minute. After intervention, the mean pulse decreased to 76.94 (SD 6.62) beats per minute, indicating a significant improvement.

### Intervention Adherence

Among the 33 participants in the intervention group, 32 (97%) provided valid data for adherence analysis. The average number of completed sessions was 37.84 (SD 9.83), with a median of 42 sessions. A total of 17 (51%) participants completed all 42 sessions, while 26 (79%) participants completed at least 30 sessions. Only 6 (18%) participants completed fewer than 30 sessions, and 1 (3%) participant had no adherence data available.

### Participant Satisfaction

Participant satisfaction with the eHealth intervention was quantitatively assessed. Of the participants in the intervention group, 87% (29/35) reported being “very satisfied” with the intervention, while 9% (3/35) were “satisfied,” and 3% (1/35) expressed a neutral stance. No participants reported dissatisfaction.

In addition, 91% (32/35) of participants found the intervention “easy to use,” and 86% (30/35) stated that it “helped them feel more supported during their pregnancy.” These quantitative measures highlight the high level of acceptability and usability of the intervention.

No serious adverse events were reported. Only mild cases of dizziness (3/35, 9%) and temporary nausea (2/35, 6%) were recorded, all of which resolved spontaneously within a few minutes without requiring interruption of the intervention.

## Discussion

### Principal Findings

This study examined the efficacy of a low-intensity eHealth intervention supported by IVR technology in improving mental health outcomes during pregnancy. The results provide evidence that the intervention contributed to significant reductions in anxiety and depression scores among participants in the intervention group, compared to the control group. Specifically, participants in the intervention group experienced a decrease in EPDS scores from 11.32 to 7.25, while the control group showed an increase from 11.32 to 16.23. A mixed-effects model revealed a significant interaction between time and group (coefficient=−9.03; *P*<.001), emphasizing the intervention’s effect on mitigating depressive symptoms. Similarly, for STAI-E, the intervention group’s score decreased from 61.89 to 35.03, whereas the control group’s score increased to 72.91. These findings underscore the intervention’s capacity to address acute and persistent anxiety symptoms during pregnancy. The reduction in EPDS and STAI-E scores could be related to changes in cortisol levels, sleep quality, or social support [38].

The choice of IVR as an intervention tool is based on the growing scientific evidence supporting its efficacy in reducing anxiety and depression. Previous studies have shown that IVR can induce states of relaxation, facilitate emotional regulation, and reduce stress through immersion in controlled environments designed to promote psychological well-being [18,19]. Its ability to isolate the user from external stimuli and generate an immersive experience promotes greater adherence to, and engagement with, the intervention, which is particularly beneficial in pregnant populations. Furthermore, previous research has shown that IVR activates neurobiological mechanisms associated with the reduction of stress and anxiety [20], reinforcing its suitability as a low-risk, nonpharmacological strategy that is easy to implement in this context. Although the intervention was not specifically validated in women from various cultural contexts, it was designed to be accessible and applicable to a broad spectrum of users. The content of the intervention focused on mindfulness and relaxation exercises, techniques widely used and recommended in perinatal mental health care. To ensure that it was accepted and understood, a neutral and accessible language was used, without specific cultural references that could limit its applicability in different settings. In addition, priority was given to usability and ease of integration into the daily routine of the participants, which are the key aspects to maximize adherence to the intervention.

Our approach in this study is based on modern theories of emotional processing, which postulate that emotional responses are mediated by neural networks associated with memory and fear regulation [39]. IVR facilitates immersion in relaxing and controlled environments that induce a physiological relaxation response, promoting regulation of the autonomic nervous system and reducing activation of the amygdala, a key structure in the response to stress and anxiety [19]. Furthermore, the immersive experience in IVR promotes attentional focus and cognitive distraction, mechanisms that have been shown to be effective in reducing symptoms of anxiety and depression [18,20]. The intervention is based on mindfulness-based therapy, which has been widely validated in the treatment of anxiety and depression [16]. IVR enhances this practice by providing an immersive environment that facilitates the experience of presence and engagement with the activity, optimizing therapeutic benefits. Repeating sessions over 6 weeks favors the consolidation of emotional regulation patterns through neuroplasticity mechanisms, generating sustained changes in the perception of stress and anxiety [21]. Thus, the theoretical model that supports our intervention combines the physiological regulation of stress through modulation of the autonomic nervous system, cognitive distraction and attentional focus facilitated by immersion, and the progressive internalization of emotional coping strategies through the repeated practice of mindfulness in a safe and structured environment.

Although our study protocol did not include a specific assessment of sleep quality or anxiety sensitivity, some publications suggest that anxiety and depression during pregnancy are closely related to these factors. Previous studies have shown that mindfulness- and relaxation-based interventions, such as the one used in our study, can improve sleep quality and reduce stress levels [40,41]. Furthermore, it has been shown that anxiety sensitivity can act as a vulnerability factor for the development of anxiety and depressive symptoms, which reinforces the relevance of addressing these aspects in future research [42].

There are several mechanisms that may explain the observed benefits of IVR-based intervention on mental health. First, the cognitive and emotional distraction effect plays a key role in reducing symptoms of anxiety and depression. The immersive nature of virtual reality allows attention to be diverted from stressors, providing a controlled and immersive environment that promotes relaxation. This mechanism has been demonstrated in previous studies, in which exposure to virtual environments effectively reduced the perception of stress and pain in clinical contexts. Second, the induction of physiological relaxation may be a determining factor. The IVR intervention includes mindfulness and guided relaxation techniques, which have been associated with reduced autonomic activation, decreased heart rate, and better emotional regulation. These effects are consistent with our findings, in which a significant reduction in symptoms of anxiety and depression, as well as a decrease in heart rate, was observed in the intervention group. Third, the influence on neuroplasticity and emotional engagement could contribute to the positive effects of IVR. Immersive experiences can modulate neural circuits associated with stress and mood regulation, promoting adaptive cognitive and emotional responses. Furthermore, the activation of reward-related brain areas during the virtual experience can improve mood and motivation, which would explain the high levels of adherence and satisfaction observed in our study. Finally, the increase in the perception of support and self-efficacy could mediate the effectiveness of the intervention. By providing a structured, engaging, and easily accessible tool for mental well-being, IVR allows users to take an active role in managing their symptoms. This sense of control has previously been linked to better outcomes in digital health care interventions. In any case, future studies should explore these mechanisms in greater depth by using neuroimaging techniques, physiological monitoring, and qualitative evaluations of the user’s experience.

Regarding the generalizability of the findings, we acknowledge that the study was conducted in a specific region of Spain, which might limit its applicability to other populations. Nevertheless, to strengthen external validity, the following 2 strategies were implemented: (1) participants were recruited from 5 primary care centers (ASSIR), allowing for the inclusion of women with different sociodemographic profiles within the public health system; and (2) the virtual reality intervention used does not depend on specific cultural or contextual factors, facilitating its implementation in other prenatal care settings. Our findings are consistent with previous studies on eHealth and virtual reality for the management of anxiety and depression, suggesting that this technology may be effective in several pregnant populations.

With regard to the representativeness of the sample and the strategies to improve diversity, participants were selected in public primary care centers, ensuring equal access to the intervention and avoiding biases associated with the use of private services. Broad inclusion criteria were established, including women aged ≥18 years with moderate symptoms of anxiety and depression (EPDS scores: 9-12), which favored the heterogeneity of the sample. All participants had to speak and understand Spanish, ensuring understanding of the intervention and evaluation of the results without linguistic bias. Although differences were observed in the educational level of the participants, we performed adjusted analyses to control possible effects of these variables on the results.

In our study, no serious adverse events were reported. A pilot study in the United States analyzed the feasibility of IVR for distraction and pain management in women in labor and reported a high acceptance with no serious side effects [43]. While in the general population the use of IVR may be associated with effects such as dizziness, nausea, or disorientation—known as cybersickness—these effects appear to depend on the design of the intervention, the duration of exposure, and the individual characteristics of the users [44]. Although the evidence so far suggests that IVR is a safe and well-tolerated intervention in pregnant women, we encourage future research evaluating potential adverse effects in the long term and in more diverse populations.

### Comparison With Previous Literature

Our findings align with previous literature emphasizing the potential of digital mental health interventions for perinatal populations. For example, Bell et al [45] demonstrated the effectiveness of virtual reality in managing anxiety and depression in diverse clinical settings. Similarly, studies such as Pallavicini et al [46] highlighted the ability of virtual reality games to elicit positive emotions and reduce state anxiety through immersive and interactive experiences. These findings suggest that IVR technology’s immersive nature enhances user engagement and emotional regulation, supporting its use as a tool for mental health interventions during pregnancy.

In addition, Fuster-Casanovas et al [47] observed a significant rise in the adoption of eHealth tools for managing mental health in Catalonia, further highlighting the role of digital platforms in enhancing accessibility to psychological support. Their findings resonate with our study’s results, as both suggest that digital tools, particularly IVR, can bridge gaps in care by offering scalable and engaging solutions for vulnerable populations. Moreover, Bell et al [48] reported that virtual reality interventions significantly reduced anxiety in adolescent patients in hospital settings when immersive, therapeutic applications were used, providing further evidence for the clinical utility of such technologies in diverse populations.

### Satisfaction

Participant satisfaction with the IVR-based intervention was remarkably high, as evidenced by postintervention surveys. Participants reported that the immersive nature of the technology facilitated relaxation and emotional engagement, which contributed to the observed reductions in anxiety and depression symptoms. These findings are consistent with previous studies, such as Bell et al [48], which demonstrated that virtual reality interventions elicited positive emotional responses and were well-received by users. In addition, Pallavicini et al [46] noted that the interactive elements of virtual reality contributed to enhanced user experiences, making the intervention more enjoyable and effective. Further support comes from Al Kuwaiti et al [49], who highlighted that virtual reality’s adaptability and immersive environments increase patient satisfaction in therapeutic applications. The positive feedback underscores the acceptability of IVR as a feasible mental health intervention, particularly in populations with limited access to traditional therapies.

### Adherence

Adherence to the intervention protocol was high, with 97% (32/33) of participants providing valid data. Among them, 79% (26/33) completed at least 30 sessions. This is in line with findings from Fuster-Casanovas et al [47] who highlighted the ease of use and accessibility of eHealth tools as key factors in ensuring consistent engagement. High adherence rates were also observed in studies using similar virtual reality–based interventions, such as Bell et al [45], which attributed participant compliance to the engaging and user-friendly nature of the technology. In addition, findings by Garcia et al [50] suggested that virtual reality interventions designed with user-centric features significantly improved adherence by maintaining user motivation and interest. Furthermore, Indovina et al [51] demonstrated that adherence to virtual reality–based pain management programs was strongly associated with the level of interactivity and the ability to tailor content to user preferences. These findings suggest that the immersive and interactive characteristics of IVR not only enhance satisfaction but also foster sustained participation, which is critical for achieving meaningful mental health outcomes.

We found that user-centered design and personalization of IVR is very important. As far as individual needs assessment is concerned, before implementing the IVR intervention, it is essential to conduct an initial assessment of each pregnant woman’s needs, preferences, and expectations. This may include questionnaires about her anxiety and depression levels, as well as her preferences regarding types of exercises (eg, mindfulness, relaxation, and guided imagery) and virtual environments (eg, beaches, forests, and mountains) [23]. This assessment would allow for the creation of a personalized intervention plan that fits each woman’s specific needs, thus increasing adherence to, and satisfaction with, the intervention [52]. As far as adaptation of virtual environments is concerned, they should be designed to be inclusive and culturally sensitive. For example, women from certain cultural backgrounds may feel more comfortable with environments that reflect their natural or cultural environment. This could involve adapting landscapes, sounds, and narratives to be culturally relevant [53]. Furthermore, environments should be adjustable in sensory intensity (eg, sounds, colors, and movement) to suit individual preferences and avoid overstimulation, especially in women who may be more sensitive during pregnancy [54]. Regarding flexibility of duration and frequency of the IVR session, they should be flexible to suit pregnant women’s routines and energy levels. Some women may prefer shorter but more frequent sessions, while others may benefit from longer but less frequent sessions [55]. The ability to pause and resume sessions is also important, as pregnant women may need frequent breaks due to physical discomfort or fatigue [56]. The incorporation of real-time feedback systems can improve the personalization of the intervention. For example, heart rate sensors or short questionnaires during sessions can provide information about the woman’s emotional state, allowing the IVR content to be adjusted based on her responses. This dynamic approach can help ensure that the intervention is always relevant and effective for each woman [56]. Finally, to ensure that the IVR intervention is truly user-centered, it is essential to involve pregnant women in the design and development process. This may include focus groups, pilot testing, and satisfaction surveys to gather direct feedback about the content and usability of the intervention [57]. Active user participation not only improves the acceptance of the intervention but also increases the likelihood that it will be effective and sustainable in the long term [58].

### Strengths and Limitations

While our study contributes to the growing evidence base for IVR interventions, it is not without limitations. First, the relatively small sample size restricts the generalizability of the findings. Second, the reliance on self-reported adherence and satisfaction data introduces potential reporting biases. In addition, the absence of long-term follow-up limits our ability to evaluate the sustained impact of the intervention on postpartum mental health.

Another limitation to consider is related to the extrapolation of these results to women from disadvantaged socioeconomic backgrounds, or whose technological limitations could be restrictive, given that the availability of, and familiarity with, technologies such as IVR can differ significantly between socioeconomic groups. Also, the representativeness of the sample is limited because the participants generally had a high educational level and good access to health care and technological resources, which could bias the results toward greater effectiveness and adherence. Another potential limitation is related to possible biases derived from self-reporting of adherence to, and satisfaction with, the intervention, which may reflect a social desirability bias. Finally, it is worth noting that long-term follow-up was not conducted, which limits conclusions about the sustainability of the treatment’s effect on postpartum mental health. Future research should therefore consider, including more diverse samples, including women from different socioeconomic and cultural backgrounds, as well as evaluating alternative methods of technological access that facilitate the equal participation of women in vulnerable situations. In addition, future studies should implement longer follow-ups to assess the sustainability of the observed impact on mental health and delve deeper into possible biases arising from self-reporting and the specific characteristics of the studied group.

Nonetheless, this study has several strengths. The use of IVR technology represents an innovative and accessible approach to perinatal mental health care. Rigorous adjustments for baseline differences strengthen the reliability of our findings, and the high adherence rates suggest feasibility for real-world implementation. Importantly, the results of this study provide a foundation for future research to build upon, particularly in exploring the long-term effects and cost-effectiveness of IVR interventions for pregnant women.

### Clinical Implications and Future Directions

From a clinical perspective, the integration of IVR-based eHealth programs into prenatal care offers a promising avenue to address gaps in mental health support. These programs can serve as scalable and engaging solutions, particularly in contexts where traditional resources are limited. Future studies should explore how to customize IVR content to meet individual needs and preferences, potentially enhancing both engagement and outcomes. Fajnerova et al [59] emphasized the potential of multiuser virtual environments for facilitating group-based mental health interventions, which could be explored further in maternal care settings.

Our primary objective in this RCT was to assess the immediate efficacy of the IVR intervention on symptoms of anxiety and depression during pregnancy, using standardized instruments (EPDS and STAI). However, the evaluation of durability of these beneficial effects beyond the immediate intervention period is essential to confirm its clinical utility and inform maternal mental health policies. We therefore plan to conduct future longitudinal studies that include extended postpartum follow-up periods (eg, from 6 to 12 months after birth) to assess whether reductions in prenatal symptoms of anxiety and depression symptoms are maintained after delivery. These future studies will adopt longitudinal designs to analyze the persistence of improvements in maternal mental health outcomes, including the potential prevention of postpartum depression (PPD) and anxiety. In addition, long-term secondary outcomes related to maternal and infant health will be studied, specifically assessing maternal psychological well-being, mother-child bonding, parenting stress, and infant development trajectory using validated tools. These assessments will help determine whether the benefits observed during pregnancy have significant long-term effects, contributing to improved mother-child interactions and improved infant development trajectories. In addition, to improve the robustness and generalizability of the findings, future studies will include larger and more diverse cohorts, multicenter trials, and economic analyses to assess the cost-effectiveness of IVR interventions and their long-term effects on postpartum mental health. We believe that this comprehensive approach will provide robust evidence about the clinical relevance, sustainability, and scalability of IVR interventions in perinatal care settings.

We are also aware that there are some limitations related to the implementation of IVR-based interventions and eHealth technologies in real-world clinical settings. Effective implementation will require rigorous compliance with data protection regulations (eg, General Data Protection Regulation), clear informed consent processes, and robust cybersecurity measures to protect confidentiality among users. Furthermore, it is critical to consider the question of equity. The sociodemographic characteristics observed in this study suggest a potential bias toward participants with higher educational levels and stable economic situations, which could limit the applicability of the intervention in socioeconomically disadvantaged populations. Addressing these equity concerns involves ensuring technological accessibility, affordability, and ease of use across diverse socioeconomic groups and geographic locations, while also considering differences in technological ability and internet connectivity. Financial constraints are also a barrier, particularly relevant in the context of national health care services, where resources are often limited and require careful budgeting. The implementation of these technologies may require considerable initial investments, as well as clear financial strategies to ensure long-term sustainability and cost-effectiveness. Finally, practical challenges, such as integration into existing health care services, obtaining acceptance by health care professionals, and devising sustainable financing models must also be considered. For all of these reasons, future research should focus on longitudinal analyses, assessing not only immediate efficacy but also sustained use and real-world feasibility, with the aim of developing scalable and inclusive solutions tailored to different clinical contexts and populations.

In relation to how to integrate IVR-based interventions into the health care system, we propose different key aspects as shown in Textbox 2.

**Proposed strategies to integrate immersive virtual reality (IVR)–based interventions into health care system.**

**Implementation within primary care and maternity services**

- Midwives and health care professionals could incorporate IVR sessions into routine prenatal visits, offering guided relaxation and mindfulness exercises as a complementary mental health support tool.
- The intervention could be integrated into antenatal education programs, in which pregnant women receive structured sessions on stress management and emotional well-being using IVR technology.

**Home-based digital health care support**

- Pregnant women could be prescribed access to the IVR application for home use, with health care professionals monitoring adherence and outcomes through a digital platform.
- A mobile app could complement the IVR sessions by providing additional psychological support, reminders, and educational content tailored to individual needs.

**Scalability within public health care system**

- Collaboration with public health care providers could facilitate the incorporation of IVR technology into maternal mental health care programs, ensuring accessibility for a broad population.
- Future research should focus on cost-effectiveness analyses and feasibility studies to assess the sustainability of implementing IVR interventions on a larger scale.

Regarding to how to implement IVR in routine clinical practice, we consider the following recommendations for the integration of IVR into antenatal care protocols:

1. Early detection of anxiety and depression symptoms: through the implementation of IVR, routine screening for anxiety and depression symptoms could begin during antenatal visits, using validated tools, such as EPDS and STAI-E. Women presenting with moderate anxiety or depression symptoms (scores within the range established in our study) could be referred for the IVR intervention as part of a stepped care approach.
2. Training of professional health care staff, especially midwives and primary care nurses, in the use of IVR technology and in supervising sessions: this would include training in setting up the equipment, explaining relaxation and mindfulness exercises, and monitoring patients’ progress. It would also be important to provide guidelines about how to address potential questions or concerns from patients regarding the use of technology.
3. Access to technology: primary care centers could have IVR equipment available at antenatal follow-up consultations. Alternatively, lending IVR devices to patients for use at home could be considered if adequate follow-up is ensured. The intervention could be designed to be self-guided, with preprogrammed sessions that patients could complete independently, but with the possibility of contacting a health care professional in case of questions or need for additional support.
4. Integration into the individualized care plan, including the IVR intervention and tailoring to each patient’s specific needs and the context of their pregnancy: this could include coordination with other mental health professionals, such as psychologists or psychiatrists, in cases where a more intensive approach is required. In addition, it could be combined with other low-cost interventions, such as prenatal exercise programs, mindfulness workshops, or support groups, to offer a holistic approach to mental well-being during pregnancy.
5. Continuous monitoring and evaluation: a continuous monitoring system should be established to evaluate the effectiveness of the intervention and adjust it as necessary. This could include collecting data about adherence, patient satisfaction, and changes in anxiety and depression symptoms over time. Health care professionals could use the data collected to identify patients who could benefit from additional or more intensive interventions.
6. Cultural and socioeconomic considerations: as accessibility and acceptance of technology may vary depending on the cultural and socioeconomic context, it is important to adapt the intervention to the specific needs of each population. This could include translating content into different languages or adapting IVR scenarios to make them culturally relevant.

We have also reflected on the potential long-term effects of the IVR intervention. In the effects on postpartum mental health, we should consider the following: first, anxiety and depression during pregnancy are known risk factors for the development of PPD [8]. Because our intervention demonstrated a significant reduction in anxiety and depression symptoms during pregnancy, it is plausible that it may also contribute to a decreased risk of PPD [60]. Previous studies have shown that interventions that improve mental health during pregnancy have a protective effect against PPD, suggesting that IVR could have a positive impact on women’s emotional well-being after delivery [61]. Second, maternal mental health is closely related to the quality of the mother-child bond [62]. A reduction in anxiety and depression symptoms during pregnancy could facilitate a stronger and more positive bond between mother and infant, which in turn could have beneficial effects on the child’s emotional and social development [6]. Future studies could assess the impact of the IVR intervention on the quality of the mother-child bond using validated scales, such as the Postpartum Bonding Questionnaire [63]. In addition, regarding the effects on child development, we can consider the following: first, maternal anxiety and depression during pregnancy have been associated with an increased risk of problems in the child’s cognitive and emotional development, including learning difficulties, attention problems, and emotional disorders [64]. By reducing these symptoms during pregnancy, the IVR intervention could contribute to a healthier development of the child [16]. Longitudinal studies could evaluate the impact of the intervention on the cognitive and emotional development of children using tools such as the Bayley Scales of Infant and Toddler Development or the Child Behavior Checklist [65]. Second, some research suggests that maternal stress and anxiety during pregnancy may be associated with an increased risk of neurodevelopmental disorders, such as attention-deficit/hyperactivity disorder or autism spectrum disorder [66]. Although the relationship is not direct, it is possible that reducing stress and anxiety during pregnancy through the IVR intervention may have a protective effect in this regard [61]. Future studies could explore this hypothesis by long-term follow-up of children whose mothers participated in the intervention. Finally, regarding future research, we consider the following: first, to assess the long-term effects of the IVR intervention, longitudinal studies following women and their children for several years after delivery would be necessary. These studies could measure not only maternal mental health, but also children’s cognitive, emotional, and social development [60]. Second, it is important to explore the underlying biological and psychological mechanisms that could explain how reducing anxiety and depression during pregnancy influences postpartum mental health and child development. For example, changes in cortisol levels or other biological markers associated with stress could be investigated [6]. Third, future research could explore the effect of combining IVR intervention with other postpartum interventions, such as parenting support programs or cognitive behavioral therapies, to maximize benefits on maternal mental health and child development [16].

We consider that feasibility, scalability, and costs are key aspects for the IVR interventions to be effectively implemented, especially in underserved populations where access to mental health care is limited. With regard to feasibility and scalability in real-world settings, we consider the following: first, the IVR intervention requires the use of virtual reality devices, which can vary in cost and complexity. In our study, we used midrange devices that are relatively affordable and easy to use. These devices could be purchased by primary care centers or maternal health clinics or even rented to reduce initial costs. IVR technology is becoming more accessible and device costs have decreased in recent years, making it easier to implement in resource-limited settings. Second, implementation of the IVR intervention would require basic training of health care personnel, such as midwives, nurses, or psychologists, in using the technology and supervising sessions. This training could be done in a short period and would not require advanced technical skills. Furthermore, the intervention could be designed to be self-guided, which would reduce the need for constant supervision by health care staff. Third, the IVR intervention is highly adaptable and could be tailored to meet the needs of various populations, including those with language or cultural barriers. For example, relaxation and mindfulness scenarios could be adapted to reflect culturally relevant environments, and content could be translated into different languages. Regarding cost and resource estimation, we consider the following:

1. Device cost: the cost of IVR devices can vary between €300 (US $327) and €600 (US $654) per unit, depending on the brand and features. For a primary care center with a moderate workload, several devices could be purchased for rotational use.
2. Content development: the development of IVR content (eg, mindfulness exercises and relaxation) may involve an initial cost, but once developed, the content can be used repeatedly without significant additional expenses.
3. Maintenance: maintenance costs are relatively low, as modern IVR devices are long-lasting and require few upgrades.
4. Training: training of health care staff could be completed in a short period, such as a one- or two-day workshop, at an estimated cost of €500 (US $545) to €1000 (US $1090), depending on the number of participants. This training would not require advanced technical skills.
5. Implementation and long-term savings: the intervention could be designed to be self-guided, reducing the need for constant supervision by health care staff. In the long term, the IVR intervention could generate significant savings by reducing the need for more expensive interventions (eg, pharmacological or individual therapies), particularly for cases of mild to moderate anxiety and depression. Additionally, improved mental health in pregnant women could help reduce obstetric and neonatal complications related to stress and depression.
6. Adaptability: the IVR intervention is highly adaptable and could be tailored to meet the needs of various populations, including those with language or cultural barriers. For example, relaxation and mindfulness scenarios could be culturally adapted and translated into different languages.

All currency conversions from euros to US dollars were calculated using the average exchange rate at the time of the study (€1=US $1.09).

In addition, by improving the mental health of pregnant women, costs associated with obstetric and neonatal complications related to stress and depression could be reduced.

In relation to scalability in underserved populations, we consider the following: First, the IVR intervention could be particularly useful in rural or remote areas where access to mental health care professionals is limited. The devices could be transported between different health facilities or even used at home under remote supervision. In addition, the portable nature of IVR devices allows their use in communities with limited infrastructures if there is access to electricity and adequate space for sessions. Second, to facilitate implementation in underserved populations, collaborations could be established with nongovernmental organizations or public health agencies already working in these areas. These organizations could provide logistical and financial support for the acquisition and maintenance of the devices.

In this study, women with a history of diagnosed anxiety or depression were excluded because this clinical subgroup could present a different evolution compared to those with recent and moderate symptoms, which could affect the validity of the results. However, we consider that future studies could evaluate the effectiveness of this intervention in populations with a previous psychiatric history. We also consider that future studies could further explore the influence of pregnancy planning on the effectiveness of digital interventions for perinatal mental health.

It is worth remembering that IVR has been applied in other areas of mental health: first, IVR has been used for posttraumatic stress disorder, in which it has proved to be an effective treatment tool, particularly in the context of exposure therapy. The technology allows for the simulation of controlled and safe environments where patients can gradually confront the stimuli that trigger their symptoms, facilitating desensitization and emotional processing [67]. Studies have shown that IVR can be particularly useful in war veterans, accident survivors, or survivors of violence, in whom exposure to virtual environments can help reduce the symptoms of reexperiencing, avoidance, and hyperarousal associated with posttraumatic stress disorder [68]. Furthermore, IVR can be combined with evidence-based therapies, such as cognitive behavioral therapy, to improve treatment outcomes [69]. Second, IVR has also shown potential in the treatment of substance-use disorders, particularly in relapse prevention. Virtual environments can simulate high-risk situations (eg, bars or parties) where patients can practice coping strategies in a safe and controlled environment [70]. In addition, IVR can be used for aversion therapy, where patients are exposed to virtual stimuli associated with substance use, which can help reduce craving and dependence [71]. Recent studies have shown that IVR can be effective in treating alcohol, tobacco, and other drug addictions; improving treatment adherence; and reducing relapse rates [72]. Third, IVR has been widely used in the treatment of specific phobias, such as fear of flying, heights, or closed spaces. Gradual exposure to these stimuli in a virtual environment allows patients to confront their fears in a controlled manner, which reduces anxiety and improves functioning [73]. Furthermore, IVR can be useful in the treatment of generalized anxiety, by providing relaxing environments and mindfulness exercises that help reduce symptoms of excessive worry and muscle tension [46]. Fourth, IVR has also shown potential in supporting individuals with autism spectrum disorders, particularly in developing social skills and reducing anxiety in social situations. Virtual environments can simulate complex social interactions, allowing patients to practice and improve their communication skills in a safe environment [74]. Fifth, IVR has been used as a complementary tool in the management of chronic pain, which is often associated with mental health disorders, such as depression and anxiety. Virtual environments can distract patients from the pain and provide relaxation techniques that improve their emotional well-being [51].

Finally, we would like to mention the integration of artificial intelligence and machine learning into IVR interventions. We believe that these new tools have great potential to improve the personalization and effectiveness of IVR. Artificial intelligence–based algorithms could allow the individualization of IVR experiences based on, for example, emotional state or therapeutic response.

### Conclusions

The findings of this study highlight the significant potential of IVR-based eHealth interventions in improving mental health outcomes during pregnancy. By reducing symptoms of anxiety and depression, the intervention demonstrated its capacity to address critical mental health challenges faced by pregnant women, aligning with the growing emphasis on integrating digital health solutions into prenatal care. This intervention, grounded in immersive and interactive technology, not only achieved significant reductions in EPDS and STAI-E scores but also fostered high adherence and participant satisfaction. These results underscore the feasibility of implementing IVR-based programs as accessible, low-intensity, mental health interventions within broader health systems. Such programs hold promise for addressing disparities in access to care, particularly for vulnerable populations or those with limited availability of traditional mental health services.

Despite these, conclusions should be interpreted with caution given the relatively small sample size, the socioeconomic and educational homogeneity of the participants, and the possible presence of biases derived from self-reporting in the assessment of adherence and satisfaction. Furthermore, the lack of long-term follow-up limits the ability to determine the sustainability of the observed benefits. Therefore, although the findings support the potential efficacy of IVR-based interventions in specific contexts, additional studies with more diverse samples and longer follow-ups are required to confirm these results and explore their applicability in different socioeconomic and cultural contexts.

Future research should aim to address these limitations while exploring the scalability, cost-effectiveness, and cultural adaptability of IVR-based interventions. In addition, longitudinal studies are needed to evaluate the sustained impact of such interventions on postpartum mental health and their potential influence on maternal and child outcomes.

## Appendix

Multimedia Appendix 1 CONSORT-EHEALTH checklist.

## Abbreviations

ASSIR: Atenció a la Salut Sexual i Reproductiva (Sexual and Reproductive Health Care)
CONSORT: Consolidated Standards of Reporting Trials
EPDS: Edinburgh Postnatal Depression Scale
IVR: immersive virtual reality
OR: odds ratio
PPD: postpartum depression
RCT: randomized controlled trial
STAI: State-Trait Anxiety Inventory
