# Supplementary material for: Immersive Virtual Reality eHealth Intervention to Reduce Anxiety and Depression in Pregnant Women: Randomized Controlled Trial
Source: JMIR Hum Factors. 2025 Apr 30;12:e71708. doi: 10.2196/71708 (PMC12079065; doi:10.2196/71708)
Supplement: Multimedia Appendix 1 [file humanfactors_v12i1e71708_app1.pdf]

CONSORT-EHEALTH Checklist:

Title: Immersive Virtual Reality eHealth Intervention to Reduce Anxiety and Depression in Pregnant Women: A Randomized Controlled Trial

Authors: Marta Jiménez-Barragan et al.

---

1. Background and Objectives

Item 1a. Identification as a randomized trial in the title.
Yes. The title explicitly states that this is a randomized controlled trial (RCT).

Item 1b. Structured summary of trial design, methods, results, and conclusions.
A structured abstract is included, outlining the trial design, methods, primary and secondary outcomes, and conclusions.

2. Introduction

Item 2a. Scientific background and explanation of rationale.
The introduction discusses the prevalence and impact of anxiety and depression in pregnant women and the need for digital health interventions, specifically immersive virtual reality (IVR), to address this issue.

Item 2b. Explanation of how the intervention addresses the problem.
The manuscript describes how the IVR-based eHealth intervention facilitates relaxation and mindfulness, reducing anxiety and depression symptoms.

3. Methods

Item 3a. Description of trial design.
The study is a two-arm, randomized controlled trial comparing an IVR-based intervention with standard antenatal care.

Item 3b. Changes to trial design after commencement.
No significant changes to the trial design were made after commencement.

Item 4a. Eligibility criteria for participants.
Pregnant women (≥18 years) with moderate anxiety and depression (EPDS scores: 9-12) at 12–14 weeks of gestation were included. Exclusion criteria included severe psychiatric disorders and previous mental health treatment.

Item 4b. Settings and locations.
The study was conducted in five primary care centers in Catalonia, Spain.

4. Interventions

Item 5. Detailed description of interventions.
The intervention group underwent daily 14-minute IVR mindfulness sessions for six weeks. The control group received usual antenatal care.

5. Outcomes

Item 6a. Primary and secondary outcome measures.
Primary outcomes: changes in EPDS and STAI scores. Secondary outcomes: satisfaction with pregnancy care and intervention adherence.

Item 6b. Any changes in trial outcomes.
No changes were made to trial outcomes after study commencement.

6. Sample Size

Item 7a. How sample size was determined.
Based on previous studies, a total of 70 participants (35 per group) were required to achieve 80% power.

Item 7b. Any interim analyses and stopping guidelines.
No interim analyses or stopping guidelines were planned.

7. Randomization

Item 8a. Method used to generate the random allocation sequence.
Randomization was performed using EPIDAT software (version 4.2).

Item 8b. Type of randomization.
Simple randomization (1:1 ratio).

Item 9. Implementation of the random allocation sequence.
Participants were randomized by an independent researcher using a computer-generated list.

Item 10. Blinding (masking).
Participants and intervention providers were not blinded due to the nature of the intervention.

8. Statistical Methods

Item 12a. Statistical methods used for primary and secondary outcomes.
Mixed-effects models, logistic regression, and ROC analysis were used.

9. Results

Item 13a. Participant flow.
A CONSORT flow diagram is included.

Item 14a. Recruitment period and follow-up.
The study was conducted between October 2021 and May 2024.

Item 15. Baseline data.
Baseline sociodemographic and clinical characteristics are presented in Table 1.

Item 16. Numbers analyzed.
All 70 participants were included in the analysis.

10. Discussion

Item 20. Interpretation of results.
The study highlights the effectiveness of IVR in reducing anxiety and depression during pregnancy.

Item 21. Generalizability of results.
Results may be generalizable to similar populations receiving antenatal care.

Item 22. Overall evidence and comparison with previous research.
Findings align with previous studies supporting IVR-based mental health interventions.

11. Other Information

Item 23. Trial registration.
ClinicalTrials.gov NCT05756205.

Item 24. Availability of data.
Data are available upon reasonable request from the corresponding author.

Item 25. Funding.
This study was funded by the Departament de Salut, Generalitat de Catalunya (PERIS Grant No. SLT006/17/249), the Official College of Nurses of Barcelona, and the Mustela Foundation.

Item 26. Conflicts of interest.
No conflicts of interest were reported.
